# Improvement in medication education in a pediatric subspecialty practice

**DOI:** 10.1186/1546-0096-8-25

**Published:** 2010-09-15

**Authors:** Aarat M Patel, Kathryn S Torok, Paul Rosen

**Affiliations:** 1 Division of Rheumatology and Clinical Immunology, University of Pittsburgh, S700 BST, 3500 Terrace Street, Pittsburgh, PA 15261, USA; 2 Division of Pediatric Rheumatology, Children's Hospital of Pittsburgh of UPMC, 4401 Penn Avenue, Pittsburgh, PA 15224, USA

## Abstract

**Background:**

The purpose of this study was to measure the impact of an educational intervention on parents of children taking methotrexate (MTX) for juvenile idiopathic arthritis (JIA).

**Methods:**

This study was conducted using a pre- and postsurvey design. The parents of 100 children with JIA taking MTX for at least 2 months were surveyed during a routine office visit. The parents completed an initial questionnaire regarding the safe use, adverse effects, and guidelines for monitoring the toxicity of MTX. An educational intervention was then administered, and an identical follow-up questionnaire was given during the next office visit. Statistical analysis using a paired *t*-test (critical *P *value < 0.05) was performed on individuals who answered both questionnaires.

**Results:**

There were 100 responses to the initial questionnaire and 67 responses to the follow-up questionnaire. The mean length of time between surveys was 2.9 ± 0.9 months. In those who completed both questionnaires, the overall correct score increased significantly from 75.8% to 93.4%, respectively (*P *< 0.0001). Individuals scored the lowest (49%) on the question that addressed MTX's impact on pregnancy and fertility.

**Conclusions:**

MTX knowledge may be less than expected in the parents of children with JIA. Brief educational interventions in the pediatric subspecialty practice can significantly affect a family's understanding of their child's medications.

## Introduction

Methotrexate (MTX) is the most commonly used disease-modifying antirheumatic drug (DMARD) for the treatment of juvenile idiopathic arthritis (JIA) [1]. It is an antimetabolite that inhibits DNA and purine synthesis, commonly used as a cytotoxic agent for childhood malignancy in various regimens. At lower doses, MTX (≤25 mg) acts as an anti-inflammatory agent used for the treatment of rheumatic disease; however, it still has potential adverse effects [2]. Adult literature demonstrates that up to 93% of those taking MTX for rheumatoid arthritis will develop at least one adverse effect [3].

The most commonly reported adverse effects are gastrointestinal (nausea and vomiting) although folic acid can be used to reduce these symptoms. Ulcerative stomatitis and alopecia also occur but are much less common. Prescribers must be cautious in using MTX for women of childbearing age because of fetal death and/or congenital anomalies in pregnancy. Bone marrow suppression may occur and result in anemia, leukopenia, neutropenia, and/or thrombocytopenia. Immune suppression may lead to opportunistic infections. MTX has been associated with elevated transaminases and potential hepatotoxicity. Renal damage, pulmonary disease and dermatologic reactions can occur as well [2,4-6].

Patients are advised to obtain routine laboratory tests to monitor for potential toxicities. These include a complete blood count, liver function tests, blood urea nitrogen, creatinine and urinalysis. Periodic liver biopsy is not recommended for toxicity monitoring as it was in the past. For some patients with pulmonary disease, a chest x-ray and pulmonary function tests are also obtained periodically; however, MTX-associated interstitial lung disease is exceedingly rare in children [1].

In pediatric rheumatology practice, every parent and age-appropriate patient receives instructions and information on MTX use at the start of therapy. These instructions can vary across practitioners and may not be repeated on follow-up visits. A previous study on adults with RA taking MTX showed that knowledge of the toxicity and safe use of MTX were significantly improved by a patient education program [3]. There is a limited amount of data on parental knowledge of MTX use and educational interventions that may improve their understanding. An observation was made that the parents of children taking MTX are not always aware of potential adverse effects, appropriate monitoring and prevention of toxicity. As a quality improvement project, the parents' knowledge of the safe use, adverse effects and guidelines for monitoring toxicity of MTX was evaluated.

The primary aim was to determine whether educational interventions, including review of a MTX questionnaire and dispersal of a MTX brochure, were effective in parental education regarding MTX use in their son or daughter with JIA. It was also determined which question was answered incorrectly most often, and therefore which topics are important to emphasize with parents regarding MTX therapy.

## Methods

This quality improvement study was conducted as a pre- and post-survey design over a six month period at the Children's Hospital of Pittsburgh Rheumatology Clinic. Patients taking MTX were identified through chart review the day of their visit. Parents of children with JIA were asked to answer a five item questionnaire during a routine outpatient visit:

1. How often should blood work be checked on a child taking methotrexate?

(a) Every 4 weeks

(b) Every 6-8 weeks

(c) Every 3 months

(d) Every 6 months

2. What are the most common side effects of methotrexate?

(a) Upset stomach and nausea

(b) Skin rash and hives

(c) Dizziness and headaches

(d) Blurry vision and forgetfulness

3. Why should blood work be checked regularly in a child taking methotrexate?

(a) Monitor for worsening of the arthritis

(b) Monitor for potential side effects

(c) For research studies

(d) Compare results to other patients with similar disease

4. Why do we prescribe folic acid?

(a) It helps with the arthritis symptoms

(b) It helps to prevent side effects of methotrexate

(c) It helps prevent the eye disease associated with arthritis

(d) It helps with morning stiffness

5. How will methotrexate affect a child's ability to have children in the future?

(a) It has no affect but will cause birth defects if taken by women at the time of conception or pregnancy

(b) It will cause problems for women who want to get pregnant many years after the drug is stopped

(c) It will cause lower sperm counts in men many years after the drug is stopped

(d) It has no consequence on fertility or pregnancy

*Correct Responses: 1(b); 2(a); 3(b); 4(b); 5(a).

The questionnaire was developed by consensus of the physicians in the division and was based on information that was thought to be essential for parents to understand regarding MTX therapy. The questions were mainly derived from a brochure provided by the Arthritis Foundation which is given to patients that are initiated on MTX therapy for rheumatic conditions. There was no pilot testing of the questions; however, some content was similar to that from a previous study conducted on adults with RA [3], providing face validity of the questions and to ensure parents' comprehension when they were asked if they understood the questions prior to answering them. The child had to have been on MTX for at least 2 months and had to meet classification for JIA according to the International League of Associations for Rheumatology to be enrolled [7]. Some patients were concomitantly using biologic agents (27%) and/or corticosteroids (5%).

A single physician (AP) reviewed the results of the questionnaire with the parents in a standardized fashion and provided literature on MTX. The literature provided was a brochure from the Arthritis Foundation from which the questions were derived. During the next routine clinic visit, a follow-up questionnaire identical to the first was given to the parents. Some patients did not have a scheduled follow-up visit in the allotted time, were taken off MTX, or had a different caregiver present at the second visit; therefore, fewer participants answered the follow-up questionnaire. The patient demographics were not significantly different between those who answered the follow-up questionnaire and those who did not. Statistical analysis with a paired *t*-test (critical *P *value < 0.05) was used to compare mean scores (pre- and post-) for those individuals who answered both questionnaires. It was also determined whether the percentage correct was different between male versus female parents, length of MTX use, and subtype of disease using the Mann-Whitney *U *test (critical *P *value < 0.05). In the few cases in which both parents came to the initial visit, only one parent answered the questionnaire.

## Results

The first 100 parents who were approached answered the initial questionnaire. Sixty-seven of the initial 100 answered the follow-up questionnaire. The patient female-to-male ratio was 3:1 with an age range of 1-21 years old (mean of 9.9 ± 4.7 and median of 10). The most common subtype of JIA was rheumatoid factor (RF) (-) polyarticular disease (39%). The range of MTX dose was 5-25 mg (mean of 17.6 ± 5.1 and median of 18), with 56% taking oral tablets and 44% receiving subcutaneous injections. The duration of MTX therapy when initially surveyed ranged from 2-96 months (mean of 24.3 ± 21.8 months and median of 18). In those who completed both questionnaires (*n *= 67), the overall score on the initial questionnaire was 75.8% ± 20.8 and the overall score on the follow-up questionnaire was 93.4% ± 10.7. The difference between these scores using a paired *t*-test was statistically significant (*P *< 0.0001). The mean length of time between questionnaires was 2.9 ± 0.9 months.

**Figure 1:**
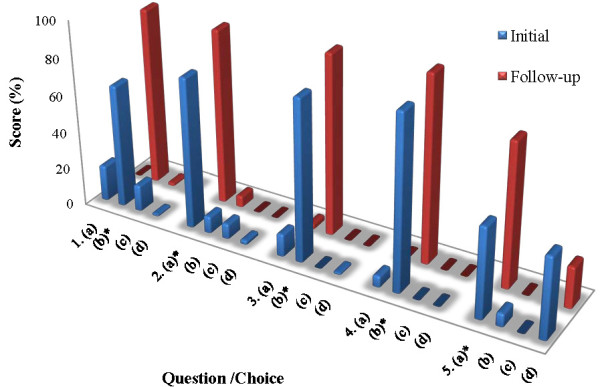
Initial and follow-up responses (n = 67).

The percentage of correct responses initially for questions 1-5 were 66%, 81%, 87%, 94% and 49%, respectively. The percentage of correct responses in the follow-up questionnaire for questions 1-5 were 95%, 94%, 97%, 100% and 77.6%, respectively. The individual question results are shown in Figure 1; all follow-up scores were significantly higher than initial scores. The lowest initial score was on question 5 (49.0%), which addressed MTX impact on pregnancy and fertility. There was no significant difference in score based on a male vs. female parent, different subtypes of arthritis or duration of MTX use (Table 1). The time points analyzed were 2-6, 7-12, 13-24, 25-36, 37-48 and over 48 months of MTX use. Some parents admitted that they did not review the MTX literature that was given to them on the initial visit. The number who actually read the literature provided was not analyzed because many patients "did not remember" if they read it.

**Table 1:** Demographics and scores by sex of parent, subtype of disease and duration of methotrexate use

Characteristic	*n *= 100 (%)	Initial	*n *= 67 (%)	Follow-Up
Sex of parent^1^				
Female	73 (73%)	77.3 ± 20.9	49 (73%)	93.5 ± 11.1
Male	27 (27%)	71.9 ± 18.6	18 (27%)	93.3 ± 9.7
Diagnosis^1^				
Oligoarticular: Persistent	34 (34%)	74.1 ± 21.2	21 (31%)	94.3 ± 11.2
Oligoarticular: Extended	5 (5%)	64.0 ± 35.8	4 (6%)	95.0 ± 10.0
Polyarticular: RF (-)	39 (39%)	81.0 ± 18.3	25 (38%)	96.0 ± 8.2
Polyarticular: RF (+)	4 (4%)	75.0 ± 25.2	3 (4%)	93.3 ± 11.5
Enthesitis-related	6 (6%)	76.7 ± 15.1	5 (7%)	88.0 ± 11.0
Psoriatic	10 (10%)	70.0 ± 14.1	7 (11%)	91.4 ± 10.7
Systemic	2 (2%)	60.0 ± 28.3	2 (3%)	70.0 ± 14.1
MTX duration, months^1^				
2-6	26 (26%)	75.4 ± 18.2	14 (21%)	95.7 ± 11.6
7-12	15 (15%)	74.7 ± 17.7	13 (20%)	89.2 ± 10.4
13-24	21 (21%	81.9 ± 18.9	13 (20%)	92.3 ± 13.0
25-36	14 (14%)	80.0 ± 24.8	9 (13%)	97.8 ± 6.7
37-48	11 (11%)	72.7 ± 20.5	9 (13%)	95.6 ± 8.8
>48	13 (13%)	66.2 ± 23.6	9 (13%)	91.1 ± 10.2

^1^The sex of parent, subtype of disease and MTX duration did not show a significant difference in questionnaire scores (Mann-Whitney *U *test used for statistical analyses). RF, rheumatoid factor; MTX, methotrexate.

## Discussion

This study demonstrated that a simple teaching intervention and distribution of literature regarding MTX significantly improved the knowledge of parents of children with JIA. The impact of this intervention was not dependent on any patient or parent characteristics, such as subtype of arthritis, length of MTX use or male or female parent. It is difficult to assess whether distributed literature or discussion regarding the questionnaire enhanced their knowledge of MTX. Both approaches to education may be important because not all individuals learn in the same way. This is not time-consuming as the questionnaire, counseling and distribution of the literature were accomplished in <10 min. The fact that the majority of parents could not recall whether they had reviewed the literature is a limitation in interpreting the effect of the literature. Another limitation is the lack of data available regarding the socioeconomic status and education level of the individuals who participated.

The results of this study emphasize that the parents of children taking MTX do not always fully understand the potential adverse effects of this medication. The majority of parents were aware of the need for monitoring laboratory tests, the common side effects of MTX and the use of folic acid. There was a lack of knowledge initially on MTX adverse effects on pregnancy (change in score from 49% correct to 77.6% correct). The average age of the patient during this study was 9.9 years old, reflecting that many were prepubertal when MTX was initiated. Healthcare workers may not have discussed pregnancy-related issues during the initiation of MTX due to the young age of the population. Another possibility is that the parents may not have been concerned about these issues because of the age of their child. Regardless of the cause, healthcare providers should consider discussing MTX and pregnancy more often and at an earlier age to establish awareness. There is limited data on children with JIA and pregnancy; however, adolescent pregnancy is always a concern, regardless of chronic disease.

A study of quality indicators showed that there was significant variability in providing adult rheumatoid arthritis patients with discussions on MTX and in obtaining appropriate monitoring studies [8]. Providing literature regarding potential medication toxicities through electronic medical records could standardize this process to decrease this variability as well as satisfy new regulatory agency requirements mandating that healthcare workers demonstrate quality of care assurance and improvement [9]. If one provided this information with the printed prescriptions, the quality indicators performance might improve; however, the patient or parent is expected to read this information to gain understanding. This does not always occur, so one still may need to provide information verbally on medications during clinic visits.

It has been recommended that educating families about goals of treatment and how to minimize treatment side effects may enhance adherence [10]. A direct correlation between improved medication knowledge and enhancing adherence cannot be made from this study but may be interesting to investigate in future studies.

In summary, there can be a lack of knowledge regarding the use and common adverse effects of MTX in JIA. The parent understands the use and toxicity of MTX can be improved with education. This can be done with a combination of verbal information by a healthcare provider and written literature on MTX. Pediatric rheumatologists should consider repetitive education when teaching families about DMARDs, which can be achieved in a short amount of time and enhance parental knowledge of MTX use and toxicity.

## Competing interests

The authors declare that they have no competing interests.

## Authors' contributions

AP distributed the questionnaires and performed the educational interventions of the study. AP and KT performed the statistical analysis. All the authors participated in the design of the study and helped draft the manuscript. All authors read and approved the final manuscript.
